# The Impact of Winter Heating on Air Pollution in China

**DOI:** 10.1371/journal.pone.0117311

**Published:** 2015-01-28

**Authors:** Qingyang Xiao, Zongwei Ma, Shenshen Li, Yang Liu

**Affiliations:** 1 Department of Environmental Health, Rollins School of Public Health, Emory University, Atlanta, Georgia, United States of America; 2 State Key Laboratory of Pollution Control and Resource Reuse, School of the Environment, Nanjing University, Nanjing, China; 3 State Key Laboratory of Remote Sensing Science, Jointly Sponsored by the Institute of Remote Sensing Applications of Chinese Academy of Sciences and Beijing Normal University, Beijing, China; Nanjing University, CHINA

## Abstract

Fossil-fuel combustion related winter heating has become a major air quality and public health concern in northern China recently. We analyzed the impact of winter heating on aerosol loadings over China using the MODIS-Aqua Collection 6 aerosol product from 2004–2012. Absolute humidity (AH) and planetary boundary layer height (PBL) -adjusted aerosol optical depth (AOD^*^) was constructed to reflect ground-level PM_2.5_ concentrations. GIS analysis, standard statistical tests, and statistical modeling indicate that winter heating is an important factor causing increased PM_2.5_ levels in more than three-quarters of central and eastern China. The heating season AOD^*^ was more than five times higher as the non-heating season AOD^*^, and the increase in AOD^*^ in the heating areas was greater than in the non-heating areas. Finally, central heating tend to contribute less to air pollution relative to other means of household heating.

## Introduction

Exposure to ambient fine particulate matter (PM_2.5_, airborne particles with an aerodynamic diameter of less than or equal to 2.5 μm) pollution has been associated with various adverse health outcomes, including cardiovascular diseases and premature death [1]. In China, approximately three times higher PM_2.5_ concentrations in winter relative to PM_2.5_ concentrations in summer have been reported by previous studies and winter heating has been identified as a main contributor to the severe PM pollution [2,3]. Coal is the main fuel used for heating and the government supported central heating system is widely used in northern China, which is driven by large-capacity boilers in heating stations and power plants. In 2010, approximately 168 million tonnes of coal was used for central heating in China [4] and a previous study reported that in Beijing coal combustion contributed 22.7 μg/m^3^ PM_2.5_ in January, in contrast with 0.7 μg/m^3^ PM_2.5_ in July in 2000 [5]. Zhang et al. [6] analyzed PM_2.5_ concentration measurements in Beijing from 2009–2010, and reported that coal combustion accounted for 18% of PM_2.5_ on an annual basis, while this source was responsible for 57% of PM_2.5_ in winter. However, these studies have two major limitations: the air quality data were only from one or several major cities and the study periods were relatively short. To fill this research gap Chen et al. [7] analyzed the variability in total suspended particulates (TSP) concentrations from 1981–2000 and reported that the ambient TSP concentrations were about 184μg/m^3^ higher in northern China than in southern China due to the central heating policy. These authors further estimated that exposure to TSP led to a reduction of 5.5 years in life expectancy at birth for the residents in the north relative to those in the south [7]. Despite the alarming results, this analysis has several limitations. First, the methods used by Chen et al. did not distinguish the impact of central heating from the impact of individual heating (e.g., stoves, electric heaters). The conclusion that the air pollution levels differ between northern and southern China as a result of the central heating policy may be inaccurate. Second, these authors grouped Chinese cities based on their locations relative to the Qin Mountains and Huai River. Although the Qin Mountains and Huai River are considered the geographical division of north and south China, they are not the dividing line between heating and non-heating areas. As we show in this analysis, this grouping may introduce substantial uncertainty in their analysis. Moreover, the air pollution data only covered several cities where the winter heating and air pollution situations may differ from those in rural and suburban regions. The estimated subsequent health impacts may not be readily generalizable to the entire Northern China. Finally, TSP is a weak indicator of PM-related adverse health effects [1,8].

The objective of this study is to examine the long-term impact of winter heating on regional particle pollution levels in China. It is unfeasible to analyze the long-term spatial trend of PM_2.5_ because ground observations covering major cities in China have only been available since 2013 and this is where remote sensing techniques open the door to analyzing long-term historical spatial trend of PM_2.5_ in China. A previous study reported that the PM_2.5_ concentrations estimated from satellite AOD through a geographically weighted regression model were highly correlated with ground PM_2.5_ concentration measurements at 50 km resolution over China, with cross-validation R^2^ values of 0.52 and 0.64 for the AOD-only model and the full model, respectively [9]. Thus, satellite-retrieved aerosol optical depth (AOD) adjusted by meteorological parameters has been used as an indicator of ground-level PM_2.5_ pollution [10,11]. The wide spatial coverage and adequate spatial resolution of satellite data allowed us to perform a comprehensive evaluation of the impact of winter heating on regional air quality over mainland China at the municipality level. We constructed a Geographic Information System (GIS) analysis to examine the temporal and spatial patterns of fine particle loadings, and developed a linear regression model to evaluate the impact of winter heating on air pollution.

## Data and Methods

### Data

**Fig 1:**
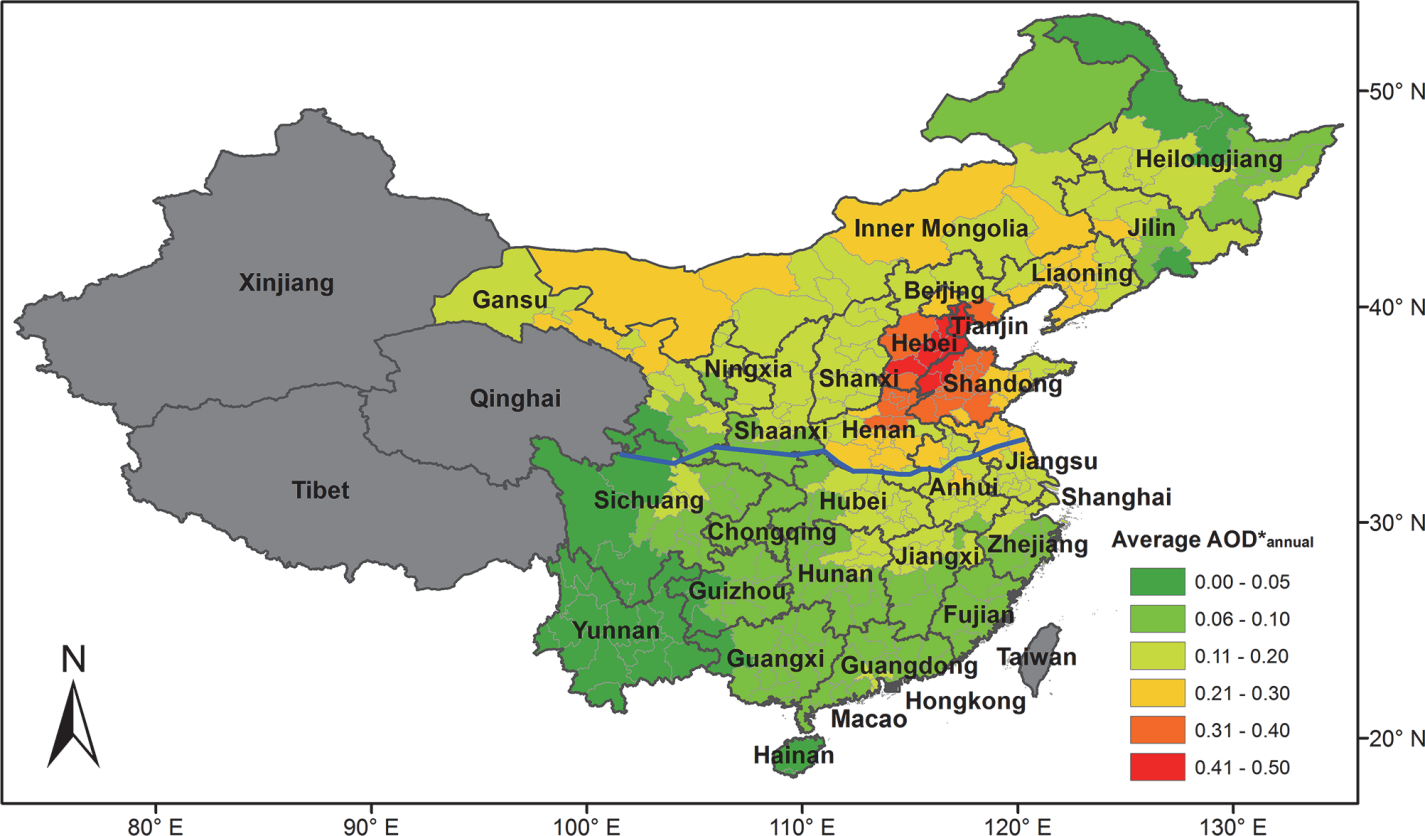
Spatial distribution of average AOD*annual from 2004–2012. The blue line along the Qin Mountains and Huai River is the traditional dividing line between north and south China. AOD^*^_annual_ varied greatly across the study domain and north China has higher aerosol loading relative to south China generally.

Our study domain covered eastern part of mainland China (Fig. 1). Tibet and Qinghai provinces were excluded because the low air temperature in these high-latitude regions requires year-round household heating, i.e., no specific heating season. Xinjiang province was excluded because frequent dust storms in this region make it difficult to distinguish the impact of anthropogenic emissions from the impact of natural sources.

The study domain includes 294 municipalities, each of which represents an average area of 20,000 km^2^. We defined the heating/non-heating seasons and areas based on the spatiotemporal coverage of central heating. The heating season, which is decided by local governments and varies yearly, generally lasts from November 16 to the end of the following February. To minimize data contamination, we excluded the days from October 15, the start date of central heating in the northernmost region of China, to November 15, the start date in southern China. March and April were also excluded from the analysis because frequent sand storms in the study region can contribute substantially to total PM levels [12]. As a result, the non-heating season was defined as between May 1 and October 14. The heating area included municipalities with operational central heating system, while other municipalities were defined as the non-heating area. The heating and non-heating areas varied slightly from year to year because the supply of central heating changed during the study period.

Collection 6 aerosol data from the Moderate Resolution Imaging Spectroradiometer (MODIS) aboard the National Aeronautics and Space Administration (NASA) Earth Observing System (EOS) Aqua satellite were obtained from the Goddard Space Flight Center (http://ladsweb.nascom.nasa.gov). We extracted the AOD parameter “AOD_550_Dark_Target_Deep_Blue_Combined” at 10×10 km nominal spatial resolution from 2004 to 2012 over China. This parameter merges AOD retrievals by the Deep Blue and Dark Target algorithms, providing high-quality retrievals with the best possible coverage [13]. The population data were obtained from the LandScan Population Project, which produces an ambient population distribution globally at approximately 1 km spatial resolution. The yearly LandScan data from 2004–2012 were obtained from the Oak Ridge National Laboratory website (http://web.ornl.gov/sci/landscan). Goddard Earth Observing System Model, version 5 (GEOS-5), produced by the NASA Global Modeling and Assimilation Office (GMAO) [14] at 0.5° x 0.667° resolution, were obtained to provide meteorological parameters, such as temperature, relative humidity, and planetary boundary layer height (PBL/km). Because individual heating activities are also common in China and also lead to air pollution, to distinguish the impact of central heating on air quality from that of total heating activities, we obtained the municipality-level central-heating data from the China Urban Construction Statistical Yearbook published by the China Planning Press.

## Methods

To account for the impact of change in humidity and vertical mixing on the association between AOD and PM_2.5_ [15], we used absolute humidity (AH g/m^3^) and PBL (km) adjusted AOD (AOD^*^) as an indicator of ground-level PM_2.5_ mass concentration (Equation 1) [11,16]. The hygroscopic growth factor (f(RH)) has been widely used by previous studies to adjust the impact of atmospheric water content on AOD [16,17]; however, due to very limited information about f(RH) in China, the large study domain with complex PM_2.5_ sources, and the long study period, it’s difficult to employ f(RH) in this study. Instead, we used AH to adjust atmospheric water content [11]. Only satellite retrievals with high or medium quality (Quality Flag = 2, 3) were included to calculate municipality-level daily average AOD^*^ and only those municipalities with at least 5% temporal coverage during both heating and non-heating seasons in each year were included to calculate the seasonal mean AOD^*^.

$$AOD^{*}=AOD/(AH\times PBL)$$ Equation 1

For each municipality, the average AOD^*^ in the heating (AOD^*^_H_), non-heating seasons (AOD^*^_N_), and the annual average AOD^*^ (AOD^*^_annual_) were calculated from the daily average AOD^*^ values to describe the PM_2.5_ pollution levels during each time period. We employed the R_AOD^*^, which is the seasonal mean AOD^*^ divided by the annual mean AOD^*^ (Equation 2), to remove the impact of interannual variability of PM_2.5_ levels and focus on their spatial contrast. To analyze the change in PM_2.5_ levels during the heating season, for each municipality, we calculated AOD^*^_diff_ and R_AOD^*^_diff_ as follows:

$$R\_AOD_{H}^{*}=AOD_{H}^{*}/AOD_{annual}^{*},\;\;R\_AOD_{N}^{*}=AOD_{N}^{*}/AOD_{annual}^{*}$$ Equation 2

$$AOD_{diff}^{*}=AOD_{H}^{*}-AOD_{N}^{*}$$ Equation 3

$$R\_AOD_{diff}^{*}=R\_AOD_{H}^{*}-R\_AOD_{N}^{*}$$ Equation 4

To control for the spatiotemporal impact of temperature on air pollution levels, two temperature variables were processed from the daily average temperature of each municipality. T_avg_ is the average of mean heating-season temperatures from 2004–2012, which reflects the spatial distribution of temperature in winter. T_var_ is the change of each mean heating-season temperature from T_avg_, which reflects the interannual variability of temperature. To better reflect the usage of central heating, the heat supply data were normalized by the corresponding population of each municipality, labeled as CentralHeat (Gcal/person).

We conducted two sample t-tests to analyze the spatiotemporal variability of AOD^*^, and then developed a linear regression model including meteorological and socioeconomic variables to explain the variability of R_AOD^*^_diff_ in China (Equation 5). In this model, the temperature variables were used as indicators of heating demands based on the assumption that heating demands are negatively related to ambient temperatures in order to maintain relatively consistent indoor temperature. Lower ambient temperatures tend to lead to greater heating demands and more heating activities. The per capital central heating term, CentralHeat, was included in this model to distinguish the impact of central heating from the impact of total winter heating on air quality. The interactions between temperature variables and CentralHeat represent additional impacts of the central heating policy on the relationship between heating demands and air pollution levels.

$$R\_AOD_{diff}^{*}\sim T_{avg}+T_{\mathrm{var}}+CentralHeat+T_{avg}\times CentralHeat+T_{\mathrm{var}}\times CentralHeat$$ Equation 5

Other predictors being considered included electricity generation and population density. Because preliminary results indicated that these variables were not statistically significant, we removed them from the final model. All data were averaged and assigned to each municipality based on their location in ArcGIS (Version 10.1; ESRI). Standard statistical tests and model fitting were conducted in SAS (Version 9.3; SAS Institute, Inc.).

## Results and Discussion

### Temporal and spatial patterns of AOD^*^

The final dataset has a total of 2,540 municipality-years. As shown in Table 1, the annual mean Aqua AOD (AOD_annual_) during the study period from 2004–2012 was 0.47, about four times higher than that typically found in North America [18]. AOD^*^_H_ (0.32) was more than five times higher as AOD^*^_N_ (0.06), and R_AOD^*^_diff_ was estimated to be 1.64 (95% CI: 1.59–1.68) over the study domain. Thus, particle loadings in the heating season were significantly higher than in the non-heating season. The variation in temperature was larger spatially than temporally: T_avg_ ranged between-19.2 ℃ in north China and 24.1 ℃ in south China, and T_var_ ranged from-3.6 to 4.3 ℃ from 2004–2012. The average CentralHeat amount was 2.1 Gcal/person, ranging from 0 (no central heating) to 55.9 Gcal/person.

**Table 1:** Summary statistics of meteorological and social-economic parameters.

	AOD_annual_	AOD^*^_H_	AOD^*^_N_	R_AOD^*^_diff_	T_avg_/℃	T_var_/℃	CentralHeat/ Gcal/person
Mean	0.47	0.32	0.06	1.64	5.3	0.03	2.1
STD	0.24	0.26	0.02	1.19	8.1	1.3	4.7
Min	0.04	-0.01	0.01	-4.09	-19.2	-3.6	0.0
Max	1.23	1.92	0.14	17.3	24.1	4.3	55.9

In Fig. 1, the spatial distribution of average AOD^*^_annual_ indicates that the AOD^*^_annual_ varied greatly across the study domain. The highest aerosol loading occurred in the North China Plain, with AOD^*^_annual_ almost three times as the national average, likely due to the high industrialization and population density in this area. A band of high AOD^*^_annual_, ranging between 0.20 and 0.26, in Gansu province and Inner Mongolia may have been produced by the dust emissions from the Gobi Desert [19]. The Sichuan Basin also had higher pollution levels relative to surrounding areas, with the AOD^*^_annual_ 10%-420% higher than the adjacent municipalities. The steady temperature inversion and stagnant air circulation in the Basin, together with high population and industrialization, potentially lead to high air pollution levels [9]. The lowest aerosol loading occurred in Yunnan, Hainan, and remote areas of Heilongjiang province due to extensive vegetation cover and low industrialization levels.

**Fig 2:**
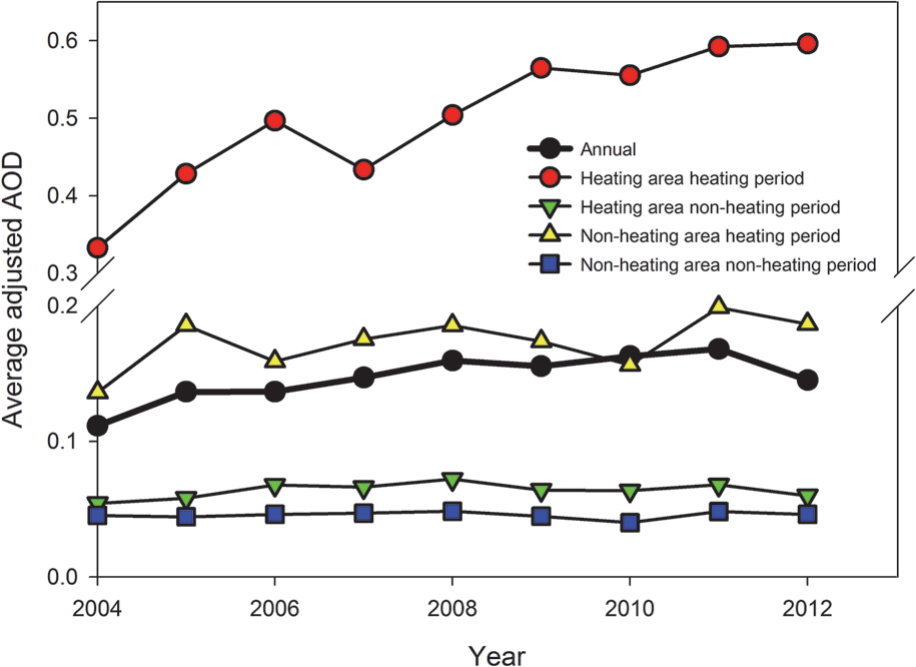
Average AOD^*^ in different spatio-temporal groups. The black line shows the AOD^*^_annual_ over the entire study region as a reference. The average AOD^*^ during the heating season in the heating area was consistently higher than other spatio-temporal groups.

Fig. 2 shows the time trend of AOD^*^ grouped by heating season and heating area. As a reference, the AOD^*^_annual_ over the entire study region increased from 0.11 in 2004 to 0.17 in 2011, and dropped to 0.15 in 2012. The AOD^*^ patterns of each group from 2004–2012 indicate that PM_2.5_ loadings during the heating season were consistently higher than the corresponding values during the non-heating season, both in the heating and non-heating areas. This increase in air pollution levels may be related to changes in meteorological conditions, such as lower wind speed and shallower boundary layer in winter, as well as winter heating [20]. During the heating season, the average AOD^*^ in the heating area was almost three times higher as in the non-heating area. The average AOD^*^ in the heating area during the heating season increased almost two fold, from 0.33 in 2004 to 0.60 in 2012, with the increase rate more than four times higher than that of the AOD^*^_annual_ over the entire study region. This PM_2.5_ pollution increase may have resulted from the increase in fuel consumption for heating, led by the increase in residents’ resources to meet their increasing heating demands. The per capita central heating area in China increased from 1.66 m^2^ in 2004 to 3.83 m^2^ in 2012 [21,22]. Previous research of PM_2.5_ sources in Beijing reported that the contribution of coal combustion to PM_2.5_ concentration during the heating season was 37% in 2000 and 57% in 2009 [6,23]. During the non-heating season, the average AOD^*^ kept steady, while the AOD^*^ in heating areas was slightly higher than in non-heating areas because of differences in energy structures in northern and southern China. Zhang et al. [24] reported that among 30 provincial capital cities, coal was the dominate energy for most cities in the north, while the southeast area relied more on electricity and oil.

**Fig 3:**
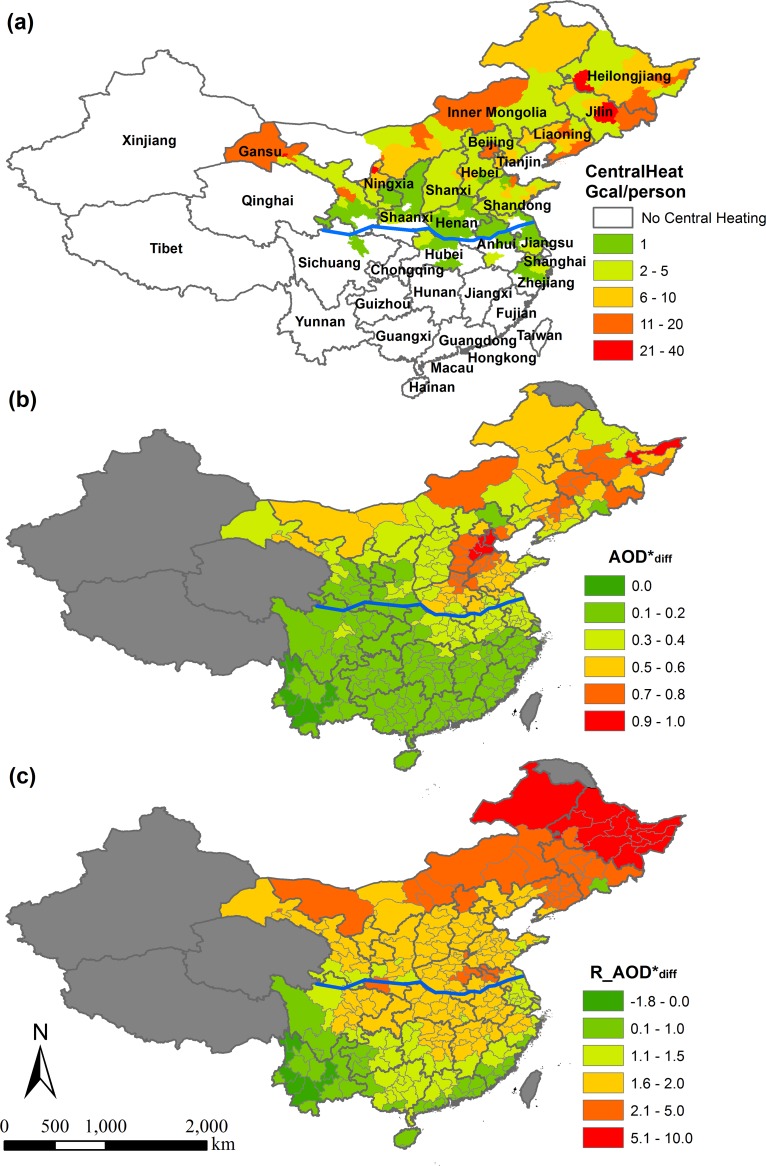
Spatial distribution of average values of parameters (a) CentralHeat (b) AOD*diff (c) R_AOD*diff from 2004–2012. The blue line along the Qin Mountains and Huai River is the traditional dividing line between north and south China.

Fig. 3 shows the spatial distribution of parameters CentralHeat, AOD^*^_diff_ and R_AOD^*^_diff_ over the study domain. The value of CentralHeat is related to the location and development status of each municipality. Well-developed municipalities in northern China often have larger CentralHeat values. Two remote areas in the north, Da Hinggan Ling Prefecture and Alxa League, have no central heating due to extremely low population density (<5 people/km^2^). Note that there are several municipalities south of the Qin Mountains and Huai River with central heating.

Results from both AOD^*^ and R_AOD^*^ indicate that PM_2.5_ levels increased significantly during the heating season over almost the whole study area (Fig. 3). A paired t-test demonstrated that during the heating season, average AOD^*^ increased significantly by 0.27 (95% CI: 0.26–0.28), 450% of the AOD^*^ during the non-heating season. The highest increase occurred in Inner Mongolia, Hebei, Heilongjiang, Jilin and Liaoning provinces, due to the colder winter and higher heating demand, while there was no such increase in some areas of Yunnan province, resulting from its warmer winter and low population density, which falls within the lowest quarter among all the provinces.

The spatial distributions of AOD^*^_diff_ and R_AOD^*^_diff_ differ due to different definitions of these two parameters. The AOD^*^_diff_ is affected by annual AOD^*^; thus, areas with high air pollution levels generally have great AOD^*^_diff_ values. In contrast, the R_AOD^*^_diff_ eliminates the annual spatial differences in AOD^*^ and emphasizes the increase ratio of air pollution levels during the heating season. The greatest increase occurs in northern part of our domain and the average R_AOD^*^_diff_ in the central heating area was almost twice as that in the non-central heating area, with a difference of 1.04 (95% CI: 0.96–1.12). During heating seasons, there were some non-random missing satellite AOD in northern China due to ice/snow coverage and this missing data may lead to an underestimation of R_AOD^*^_diff_ in northern China. In more than three-quarters of the study area, including provinces without central heating and south of the Qin Mountains and Huai River including Hunan, Guizhou, and Jiangxi provinces, AOD^*^ increased by at least one and half times as the AOD^*^_annual_ during the heating season. This spatial difference in the increase of air pollution levels during the heating season indicates that winter heating contributed to the severe air pollution in China and the impact of winter heating extended to non-heating areas. The long-range transport of PM led by the continental northeast monsoon system may contribute to this extended impact of winter heating [25].

### The Impact of Winter Heating on Air Quality

Table 2 shows the estimated regression coefficients in Equation 5. A total of 2,540 municipality-years were used to develop the model. None of the parameters in the model have a variance inflation factor (VIF) larger than 10; thus, collinearity is not a significant issue [26]. The R^2^ of the model is 0.51, meaning that our model explains more than half of the variability in R_AOD^*^_diff_. Both T_avg_ and T_var_ are statistically significant predictors, and inversely related to the increase in air pollution level (p-value <0.001). The negative regression coefficients are expected as low temperatures mean higher heating demand, leading to more pollution emissions.

**Table 2:** Estimates of parameters in the linear regression models.

	Intercept	T_avg_/℃	T_var_/℃	CentralHeat/ Gcal/person	CentralHeat×T_avg_	CentralHeat×T_var_
Estimate	2.04^a^	-0.08^a^	-0.08^a^	-0.04^a^	-0.01^a^	-0.01^a^

^a^p-value< 0.01

The parameter, CentralHeat, is inversely related to R_AOD^*^_diff_, i.e., increase in CentralHeat leads to lower air pollution levels during the heating season. The negative coefficients of the interaction terms indicate that when the temperature during the heating season is held constant, increase in CentralHeat in a given municipality will decrease its air pollution level. In other words, the central heating system has a negative effect on the association between heating demands and air pollution levels. These results suggest that the central heating system contributes less air pollution relative to other heating activities, because more efficient, centralized emission control technologies are applied in central heating system and lead to less air pollutant emission relative to fugitive emissions from individual heating devices. About 45% of the heat in the central heating system is from power plants and more than 96% of the coal-fired power plants in China installed electrostatic precipitators (ESP), which has a collection efficiency of more than 90% for PM_2.5_ [27,28]. Moreover, coal-fired power plants with capacities larger than 300MWe are required by law to install flue gas desulfurization (FGD) facilities, which is estimated to reduce SO_2_ emission by 80% [27]. Thus, higher percentage of winter heating supplied by central heating will lead to lower air pollution levels, holding total heating demands constant.

Our study provides further analysis of the impact of winter heating on air pollution in China. By using more accurate data and choosing PM_2.5_ as the target air pollutant, our results indicate that winter heating affects the air quality over a much larger area than reported previously by Chen et al. [7]. The PM_2.5_ levels during heating season increased over almost the entire domain and the population affected by the increased air pollution in the heating season exceeded 800 million in 2012. Winter heating, rather than the central heating policy, contributes largely to the severe air pollution in China and central heating has pollution-control effect relative to other household heating methods.

The relationship between AOD and PM_2.5_ is a function of particle size distribution, composition, and vertical distribution, and may vary in space and time [29]. Due to the lack of long-term PM_2.5_ measurements, we assumed that the relationship between AOD^*^ and PM_2.5_ was linear and used AOD^*^ ratio (R_AOD^*^) rather than AOD^*^ as the PM_2.5_ indicator to eliminate the impact of land use on the relationship between AOD and PM_2.5_. Thus, we analyzed the temporal variability of PM_2.5_ concentration through analyzing the R_AOD^*^. A more sophisticated statistical model with additional meteorological and land use variables is needed to better control the AOD-PM_2.5_ relationship [9,10]. Such a model, which requires multi-year PM_2.5_ measurement records, is beyond the scope of this analysis.

Although our processing of MODIS AOD data using Equations 2–4 aimed at removing interannual trends in AOD values and emphasizing the spatial contrast of PM_2.5_ levels during heating and non-heating seasons, our regression model did not fully capture other potentially important factors that can influence the seasonal variability of PM_2.5_ in China. For example, Zhang et al. [30] reported that the Asian summer monsoon can reduce PM_2.5_ levels over eastern China by 50–70% as the concentration in July is compared to that in January. In addition, more precipitation during summer months in northern China can also decrease PM_2.5_ levels because of wet deposition. A comprehensive analysis of these factors is beyond the scope of this work.

## Conclusions

By using more accurate data and choosing PM_2.5_ as the target air pollutant, this long-term and large-scale study indicates that winter heating plays an important role in increased air pollution levels in winter over almost the entire central and eastern China, and this increase is significantly higher in the heating area than in the non-heating area. The average AOD^*^ during the heating season (0.32) is more than five times higher as during the non-heating seasons (0.06), and the increase of AOD^*^ in the heating areas is almost twice higher than in the non-heating areas. Central heating has the pollution control effect relative to other household heating methods and increase in central heating leads to decrease in the PM pollution in the heating season. Our findings suggest that adopting pollution control facilities in power plants and heating stations are needed to improve winter air quality in China. Furthermore, developing heating systems supplied by renewable energy may be another sustainable solution for air quality improvement.
